# Evidence for a More Disrupted Immune-Endocrine Relation and Cortisol Immunologic Influences in the Context of Tuberculosis and Type 2 Diabetes Comorbidity

**DOI:** 10.3389/fendo.2020.00126

**Published:** 2020-03-20

**Authors:** Rocío D. V. Fernández, Ariana Díaz, Bettina Bongiovanni, Georgina Gallucci, Diego Bértola, Walter Gardeñez, Susana Lioi, Yésica Bertolin, Romina Galliano, María L. Bay, Oscar Bottasso, Luciano D'Attilio

**Affiliations:** ^1^Instituto de Inmunología Clínica y Experimental de Rosario CONICET-UNR, Rosario, Argentina; ^2^Facultad de Ciencias Médicas, UNR, Rosario, Argentina; ^3^Hospital Provincial del Centenario, Rosario, Argentina; ^4^Servicio de Neumonología, Hospital Provincial del Centenario, Rosario, Argentina; ^5^Laboratorio Central, Hospital Provincial del Centenario, Rosario, Argentina; ^6^Servicio de Medicina Transfusional, Hospital Provincial del Centenario, Rosario, Argentina

**Keywords:** pulmonary tuberculosis, diabetes mellitus type 2, immune-endocrine alterations, cortisol, glucose

## Abstract

Pulmonary tuberculosis (PTB), caused by *Mycobacterium tuberculosis* (*Mtb*), is a major health problem worldwide, further aggravated by the convergence of type 2 diabetes mellitus (DM) which constitutes an important risk factor for TB development. The worse scenario of patients with PTB and DM may be partly related to a more unbalanced defensive response. As such, newly diagnosed PTB patients with DM (TB+DM, *n* = 11) or not (TB, *n* = 21), as well as DM (*n* = 18) patients and pair matched controls (Co, *n* = 22), were investigated for the circulating immuno-endocrine-metabolic profile (ELISA), along with studies in peripheral blood mononuclear cells (PBMC) analyzing transcript expression (RT-qPCR) of mediators involved in glucocorticoid functionality. Given the hyperglycemic/hypercortisolemic scenario of TB+DM patients, PBMC were also exposed to stress-related cortisol concentrations (0.1 and 1 μM) and supraphysiologic glucose doses (10, 20, and 40 mM) and assessed for the specific response against *Mtb* stimulation (lymphoproliferation, -thymidine incorporation-, and cytokine production -bead-cytometry). All TB patients displayed increased plasma amounts of cortisol, growth hormone -hGH-, and proinflammatory mediators. In turn, TB+DM showed even higher levels of interferon gamma -IFN-γ- and hGH (vs. TB), or IL-6, C reactive protein, cortisol and hGH (vs. DM). Both DM groups had equally augmented values of IL-10. All TB patients showed decreased dehydroepiandrosterone- sulfate concentrations, even more in TB+DM cases. Leptin was also decreased in both TB cases, particularly in the TB group, revealing a lower body mass index, as well. Unlike PBMC from TB cases showing a decreased relationship between the glucocorticoids receptor (GR) isoforms (GRα/GRβ; functional isoform/negative isoform), cells from TB+DM patients had no changes in this regard, along with an increased expression of 11-beta hydroxysteroid dehydrogenase type-1, the enzyme facilitating intracellular cortisone to cortisol conversion. TB+DM patients also showed an increased *Mtb* antigen-driven lymphoproliferation. Compared to TB, DM and HCo counterparts, PBMC from TB+DM patients had a biased Th1 response to *Mtb* stimulation (increased IL-2 and IFN-γ production), even when exposed to inhibitory cortisol doses. TB+DM patients show a more unbalanced immuno-endocrine relationship, respect the non-diabetic counterparts, with a relative deficiency of cortisol immunomodulatory influences, despite their more favorable microenvironment for cortisol-mediated immune effects.

## Introduction

Pulmonary tuberculosis (PTB) is a major health problem around the world and the leading cause of death due to a pathogen, *Mycobacterium tuberculosis* (*Mtb*). In 2017, WHO reported 10 million new PTB cases, 15% of them attributed to the TB-type 2 diabetes mellitus (TB-DM) comorbidity (1). Tuberculosis clinical manifestations result from a complex interaction between its etiologic agent, and the defensive reactions developed to control infection, but its proper basis is not fully understood (2). Endocrine disturbances are likely to contribute to disease pathology, since DM increases more than three times the possibility of developing active PTB, which otherwise develop in 5–10% of *Mtb*-infected individuals. In addition to this increased risk, PTB patients with concomitant diabetes are at higher rates of treatment failure and death (3–6). Within this setting, we have recently demonstrated that patients with TB+DM showed a more pronounced adverse immune-endocrine profile than those with PTB alone (TB group) (7), for instance higher circulating amounts of cortisol.

Besides its metabolic functions, cortisol acts as an extrinsic regulator of the immune response (IR) inhibiting, at supraphysiologic concentrations, the proinflammatory response as well as the specific cellular IR against *Mtb* (8). Glucocorticoids (GC) are known to reduce the production of various cytokines such as tumor necrosis factor alpha (TNF-α), interleukins, as well as inflammatory enzymes, e.g., cyclooxygenase 2 and inducible nitric oxide synthase (9). Most immunological effects of GC are mediated by GRα isoform, whereas GRβ lacks the ability to bind GC and seems to function as an inhibitor of GRα-mediated transcriptional activation through the formation of GRα/GRβ heterodimers (10). It follows that this ratio may be related with GC functionality.

While increased susceptibility to PTB in DM patients may be linked to alterations on macrophage and lymphocyte functions (11, 12), partly related to a higher cortisol production, other factors like hyperglycemia and insulin resistance are also likely to account for such detrimental influence (3). Several studies indicate that diabetes and hyperglycemia coexist with impaired innate immune responses like phagocytosis, cytokine secretion and macrophage activation (13–17). Nevertheless, in a more recent *in vitro* study, Lachmandas et al. provided evidence that hyperglycemia failed to affect the functional capacity of macrophages against *Mtb* while being able to increase cytokine production upon mycobacterial and LPS stimulation (18).

Given this background, in the present study we sought to analyze various systemic mediators involved in the immuno-endocrine-metabolic interrelation: IFN-γ, IL-6, IL-10, IL-4, IL-1β, cortisol, dehydroepiandrosterone-sulfate (DHEA-S), prolactin, growth hormone (hGH), adiponectin, and leptin. In addition, we also quantified the expression levels of transcripts, related to the GC activity (α and β isoforms of the GC receptor -GR-), and the 11 beta hydroxysteroid dehydrogenase type 1 (11βHSD1) and type 2 (11βHSD2) enzymes. Experiments were also carried out to analyze whether high doses of cortisol (mirroring a stressful situation) along with physiological and supraphysiological glucose concentrations, or not, modified the *Mtb*-induced response of peripheral blood mononuclear cells (PBMC) from patients with TB+DM when compared to the ones yielded by TB cases, patients with DM and sex and age matched controls (Co). Assessments included lymphoproliferation studies and production of pro and anti-inflammatory cytokines as well as the Th1/Th2/Th17 profiles.

## Materials and Methods

### Sample Population

Patients (14 females and 18 males) with no HIV co-infection and newly diagnosed PTB were included. Diagnosis was based on clinical and radiological examinations together with the identification of *Mtb* bacilli in sputum. Eleven of these patients were diagnosed as also having DM (TB+DM) and 21 with only PTB (TB). For DM diagnosis, the criteria of the American Diabetes Association of 2009 were considered. Those were hyperglycemia (based on two fasting glucose levels >125 mg/dL or a random glucose level equal to or higher than 200 mg/dL) evaluated on EDTA-anticoagulated blood specimens.

The control groups were composed of 22 pair matched Co and 18 individuals with DM, sharing the same socioeconomic conditions of TB patients, unexposed to TB patients, with no clinical or radiological evidence of PTB. Patients and Co had no other respiratory disease, nor immune-compromising diseases.

The control group (Co) was composed of 22 individuals. Another group of 18 patients with DM was also included for comparison purposes. All of them were sex- and age-matched and shared the same socioeconomic conditions of TB patients, in addition to being unexposed to TB patients, with no clinical or radiological evidence of PTB. All volunteers had no other respiratory disease, nor immune-compromising diseases.

Participants were sampled by applying a consecutive non-probabilistic approach. As such, the Co group had a median BMI which fell within the range of overweight, as defined by WHO.

Blood samples were obtained on study admission, in the case of TB patients immediately before the initiation of anti-tuberculosis treatment. Samples were collected at 8 a.m. to avoid differences due to circadian variations. Exclusion criteria included disease states affecting the adrenal glands or the Hypothalamus—Pituitary- Adrenal (HPA) axis, corticosteroid treatment, pregnancy, and age below 18 years. The body mass index (BMI) was also calculated (weight/square of height). The Bioethical Committee of the School of Medical Sciences, National University of Rosario, approved the protocol. All participants gave their consent to participate in the study.

### PBMC Isolation and Lymphoproliferation

Plasma and PBMC were obtained from fresh EDTA-treated blood. Samples were centrifuged at 2000 rpm during 30 min and plasma was collected and stored at −20°C. The buffy coat was separated and diluted 1:1 in RPMI 1640 (PAA Laboratories GmbH, Austria), containing standard concentrations of L-glutamine, penicillin, and streptomycin (culture medium, CM). The cell suspension was layered over a Ficoll-Paque Plus gradient (density 1.077, Amersham Biosciences, NJ, USA), and centrifuged at 400 g for 30 min at room temperature (19–22°C). PBMC recovered from the interface were washed three times with CM and resuspended in CM containing 10% heat-inactivated pooled normal AB human sera (PAA Laboratories GmbH, Germany). Cells were cultured in quadruplicate in flat-bottomed microtiter plates (2 × 10^5^ cells/well in 200 μl) with or without addition of γ-irradiated H37Rv *M. tuberculosis* strain (*Mtbi*; 8 μg/ml, Colorado University, USA). Concanavalin A (ConA; 2.5 μg/ml, Sigma-Aldrich) was used as a proliferation positive control. PBMC cultures were incubated for 5 days at 37°C, in a 5%, CO_2_ humidified atmosphere and pulsed with ^3^H-thymidine for 18 h before cell harvesting. The average counts per minute (cpm) of stimulated and non-stimulated cultures were calculated.

### Quantification of Cytokines and Hormones in Plasma

Plasma levels of cytokines: IFN-γ (BD Pharmingen, detection limit-DL: 4.7 pg/ml), IL-10 (BD Pharmingen, DL:3.9 pg/ml), IL-6 (DRG Diagnostics, DL: 2 pg/ml), IL-4 (BD Pharmingen, DL: 7.8 pg/ml), IL-1β (BD Pharmingen, DL: 3.9 pg/ml), and hormones like Cortisol (DRG Diagnostics, DL: 2.5 ng/ml), DHEA-S (DRG Diagnostics, DL: 0.108 ng/ml), prolactin (DRG Diagnostics, DL: 0.35 ng/ml), hGH (DRG Diagnostics, DL: 0.17μIU/ml), adiponectin (Invitrogen, DL: 100 pg/ml), and leptin (Invitrogen, DL: 3.5 pg/ml) were assessed by commercial enzyme immune analysis according to the manufacturer instructions. C reactive protein (CRP) levels were measured by high-sensitivity Turbitest (Wiener Lab, DL: 2.5 mg/l). All samples were processed individually and assayed in duplicate.

### Studies on the *In vitro* Effects of Cortisol and Glucose

PBMC were cultured as above described (section PBMC Isolation and Lymphoproliferation) with the addition of various concentrations of cortisol (0.1 μM y 1 μM) (19) and/or D-Glucose (Glc; physiological dose−5 mM- and supraphysiological doses−10, 20, 40 mM- Sigma Aldrich) and further stimulated with *Mtbi*, or not. PBMC were seeded in 96-well flat-bottomed microtiter plates for the lymphoproliferation assay as previously described (section PBMC Isolation and Lymphoproliferation). The same protocol was performed in 24-well flat-bottomed microtiter plates (1 × 10^6^ cells/well). Supernatants were obtained, fractionated and preserved at −20°C until the assessment of different cytokine patterns by means of the Cytometric Bead Array Kit (LTCD4+ cells, CBA).

### RNA Isolation, cDNA Synthesis and qPCR

Total RNA was isolated from PBMC using TRIreagent (Genbiotech). RNA pellets were dissolved in Diethyl pyrocarbonate (DEPC) sterile water and stored at −80°C. RNA quantity and integrity was assessed as reported earlier (20). cDNA was synthesized from 2 μg of total RNA by extension of oligodT primers with M-MuLV reverse transcriptase (Thermo) in a final volume of 40 μl DEPC sterile water. cDNA was stored at −80°C until use. qPCR was performed with the StepOnePlus (96-well) Real-Time PCR Systems (Applied Biosystems) using 3 μl of cDNA dilution, 0.4 μM of each primer and 3 μl of 5x HOT FIREPol® EvaGreen qPCR Mix Plus (ROX) (Solis BioDyne), final volume of 15 μl. Thermal cycling conditions were as follows: 10 min at 95°C followed by 45 PCR cycles of denaturing at 95°C for 20 s, 30 s for annealing at 60°C and 30 s for elongation at 72°C. Fluorescence readings were performed during 10 s at 80°C before each elongation step. To normalize the expression of every gene, the transcript of peptidylprolyl isomerase A was used as an endogenous control on each mononuclear cell sample (21). Serially diluted cDNA samples synthesized from Jurkat and NCI-H295R cell line expressing GRα, GRβ and 11βHSD1 and 11βHSD2 mRNA, respectively (22, 23), were used as relative external standards curve in each run, to make “The Relative Standard Curve Method” for the relative quantification of gene expression, as performed formerly (20). Similarity and homogeneity of PCR products from samples were confirmed by automated melting curve analysis (StepOne Software, Applied Biosystems), revealing melting temperature values of the PCR products. Primers were designed as described by D'Attilio et al. (24), Table 1. Data were expressed as fold change of the relative expression levels of the gene of interest (GOI) normalized by the relative expression levels of PPIA.

**Table 1:** Sequence of qPCR primers.

**Transcript**	**Forward Primer**	**Reverse Primer**	**Size**
CycA ***PPIA*** **GeneID:**5478	*CycA-F* 5′-gca tac ggg tcc tgg catc ttg-3′	*CycA-R* 5′-tgc cat cca acc act cag tct tg-3′	101pb
GRα ***NR3C1***, **GeneID:**2908 **Transcript variant 1**	*GR-F* 5′gaa gga aac tcc agc cag aac-3′	*GRα-R* 5′-gat gat ttc agc taa catc tcg-3′	159bp
GRβ ***NR3C1***, **GeneID:**2908 **Transcript variant 6**	*GR-F* 5′-gaa gga aac tcc agc cag aac-3′	*GRβ –R* 5′-tga gcg cca aga ttg ttg g-3′	144 bp
11βHSD1 ***HSD11B 1***, **GeneID:**3290	*11βHSD1 F* 5′- atg ata ttc acc atg tgc gca−3′	*11βHSD1 R* *5*′- *ata ggc agc aac cat tgg ata ag-3*′	158pb
11βHSD2 ***HSD11B 2***, **GeneID:**3291	*11βHSD2 F* *5*′*-tcg cgc ggt gct cat cac-3*′	*11βHSD2 R* 5′- gta cgc agc tcg atg gca cc-3′	132pb

*PPIA, peptidylprolyl isomerase A; NR3C1, nuclear receptor subfamily 3 group C member 1; GR, glucocorticoids receptors; HSD11B1, hydroxysteroid 11-beta dehydrogenase 1; HSD11B2, hydroxysteroid 11-beta dehydrogenase 2*.

### Cytometric Bead Array

Cytokine quantification was done using the Human Th1/Th2/Th17 CBA kit (BD Biosciences) which allowed the simultaneous detection of IL-2, IL-4, IL-6, IL-10, TNF-α, IFN-γ, and IL-17A (DL: 2.6; 4.9; 2.4; 4.5; 3.8; 3.7 and 18.9 pg/ml, respectively). CBA analysis was performed according to the manufacturer's instructions. Cytokine supernatant levels were calculated using BD CBA software (version 4.0, BD Biosciences).

### Statistical Analysis

Comparisons between groups were made by non-parametric methods: Kruskall–Wallis followed by *post-hoc* comparisons when applicable. Qualitative variables were compared by the chi square test. Related samples were analyzed by means of Wilcoxon and Friedman tests. Associations between variables were analyzed using the Spearman correlation test. A value of *p* < 0.05 was considered as statistically significant.

## Results

### General Features of Study Groups

Table 2 shows the general characteristics from the different study groups and diabetes-related biochemical findings. A lower percentage of BCG vaccination was seen in the DM and TB+DM groups with respect to Co, with the TB+DM group showing the lowest percentage of a BCG scar. The TB group showed a significant decrease in the BMI if compared to Co and TB+DM. Patients with DM showed the highest BMI, but their values did not differ from the ones recorded in Co counterparts. Mainly because, by chance, 23% of Co volunteers presented a BMI within the normal weight range, whereas 55% and a 22% of them had values that fell in the overweight and obesity categories, respectively. Regarding diabetes-related biochemical findings (Table 2), DM and TB+DM groups had elevated blood glucose levels (much higher in the latter group). The percentage of glycosylated hemoglobin (HbA1c) was higher in both groups of DM patients more pronounced in those with the TB+DM comorbidity. Basal insulinemia was found increased only in the TB+DM group, statistically different from the remaining groups. When calculating the HOMA index (≥3.2 compatible with insulin resistance), 67% of DM patients and 73% of TB+DM fell in this category. It is worth reminding that most DM were under treatment with metformin and fenofibrate. In addition, the erythrocyte sedimentation rate (ESR) was increased in both groups of patients with TB in relation to their respective controls, while the DM group differed from the Co-individuals (Table 2).

**Table 2:** Main features of subjects participating in the study.

**Parameters**	**Co** **(*n* = 22)**	**DM** **(*n* = 18)**	**TB** **(*n* = 21)**	**TB+DM** **(*n* = 11)**	**Overall *p-*value**
Age (years)	45 (35–62)	54 (51–61)	41 (34–59)	50 (41–62)	n.s.
Sex (F/M)	9/13	10/8	8/13	6/5	n.s.
BCG (%)	95.4	78.9	76.2	45.5^*^^#^^*&*^	0.001
BMI (kg/m^2^)	27.0 (25.9–29.6)	28.0 (25.1–31.5)	20.9 (19.4–23.9)^*^	26.9 (23.4–31.9)^&^	<0.0001
Glycemia (mg/dl) [r.v: 70–100]	89.5 (84.3–106)	111^*^ (94.5–129)	87.0 (81.0–104)	213^*^^,^ ^#^^,^ ^&^ (161–285)	<0.0001
HbA1_C_ (%) [r.v: 4.8-6]	5.5 (5.0–5.8)	7.5^*^ (6.0–10)	5.8 (5.4–6.3)	9.7^*^^,^ ^&^ (7.0–11)	<0.0001
Insulin (μIU/ml) [r.v: 2.6-25]	6.7 (5.4–9.8)	7.4 (5.7–13)	7.0 (3.9–8.6)	14^*^,^&^ (6.4–29)	0.0003
HOMA_IR_ [r.v: ≤ 3.2]	1.43 (1.08–2.12)	3.22^*^ (1.32–4.45)	1.51 (0.832–2.20)	6.51^*^^,^ ^#^^,^ ^&^ (2.31–15.8)	0.0003
ESR (mm/1° hs) [r.v: 1-15]	5.0 (2.3–9.3)	10.0^*^ (7.8–20.5)	57.5^*^ (34.5–89.5)	60.0^*^,^#^ (36.0–92.0)	<0.0001

Co, controls; DM, patients with type 2 diabetes mellitus; TB, patients with pulmonary tuberculosis; TB+DM, patients with pulmonary tuberculosis and type 2 diabetes mellitus; n.s., not significant; F, female; M, male; BCG, Bacillus Calmette-Guerin vaccination; BMI, body mass index. rv, reference values; HOMA_IR_ index, [insulin (μIU/ml)^*^glycemia (mmol/L)]/22.5; ESR, Erythrocyte Sedimentation Rate. Results are shown as median (25-75 percentiles).

* different from HCo, p < 0.05;

# different from DM, p < 0.05;

& *different from TB, p < 0.05*.

### Plasma Quantification of Immunologic Mediators and Hormones

#### Plasma Levels of Pro and Anti-inflammatory Cytokines, Adiponectin, Leptin, Prolactin and hGH

**Figure 1:**
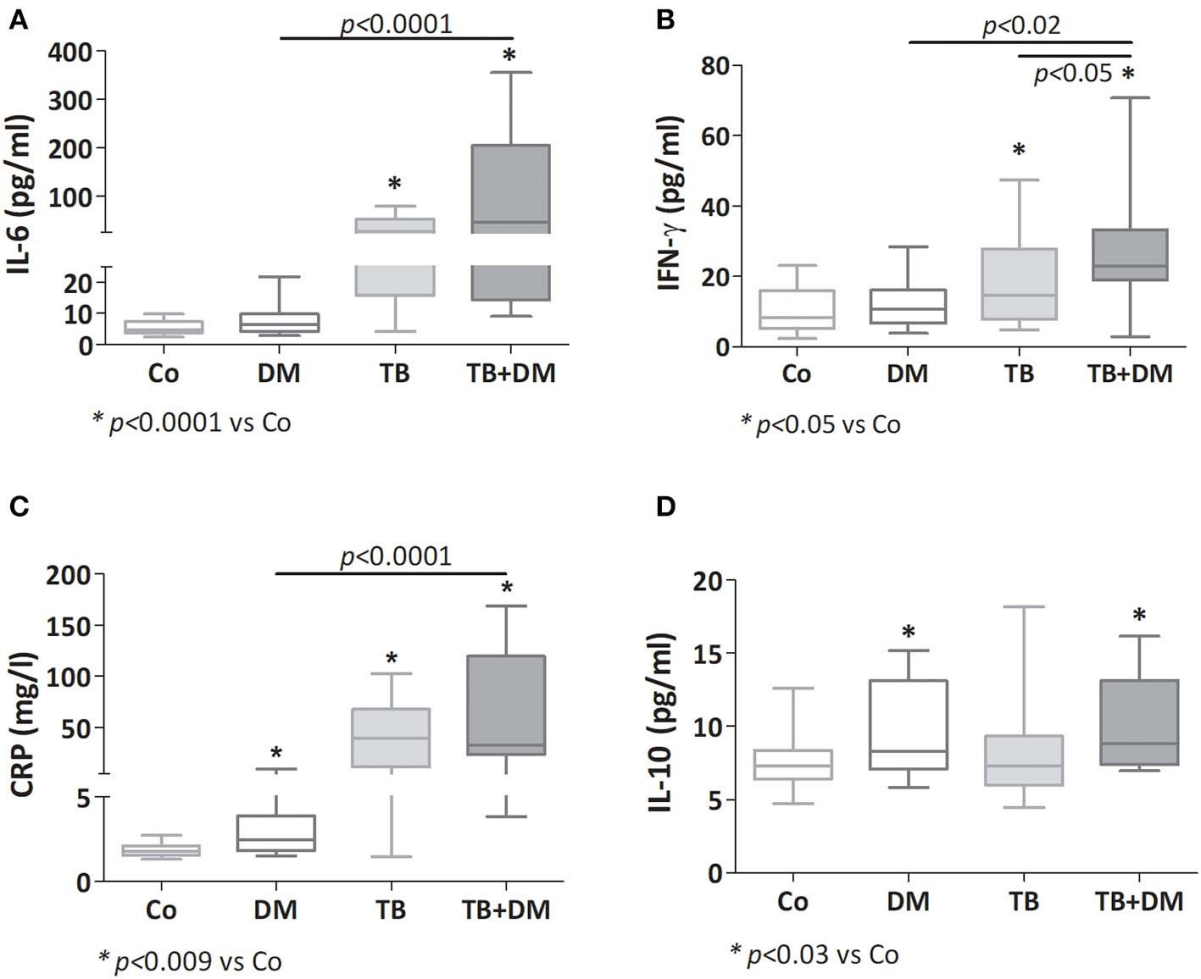
Plasma levels of IL-6 **(A)**, IFN-γ **(B)**, C reactive protein (CRP; **C**) and IL-10 **(D)** in controls (Co), patients with type 2 diabetes (DM), with pulmonary tuberculosis (TB) or with TB and DM (TB-DM). Box plots show median values, 25–75 percentiles from data in each group with maximum and minimum values.

Both groups of TB patients showed elevated values of IL-6, CRP, and IFN-γ (Figures 1A–C), with TB+DM patients showing even higher amounts of IFN-γ if compared to TB counterparts. Patients with DM also displayed increased CRP levels. As regards IL-10, this cytokine was increased in both groups of DM patients (Figure 1D), as well as in the group of TB patients with severe disease (data not shown). Assays for IL-4 and IL-1β yielded values below the detection limits.

**Figure 2:**
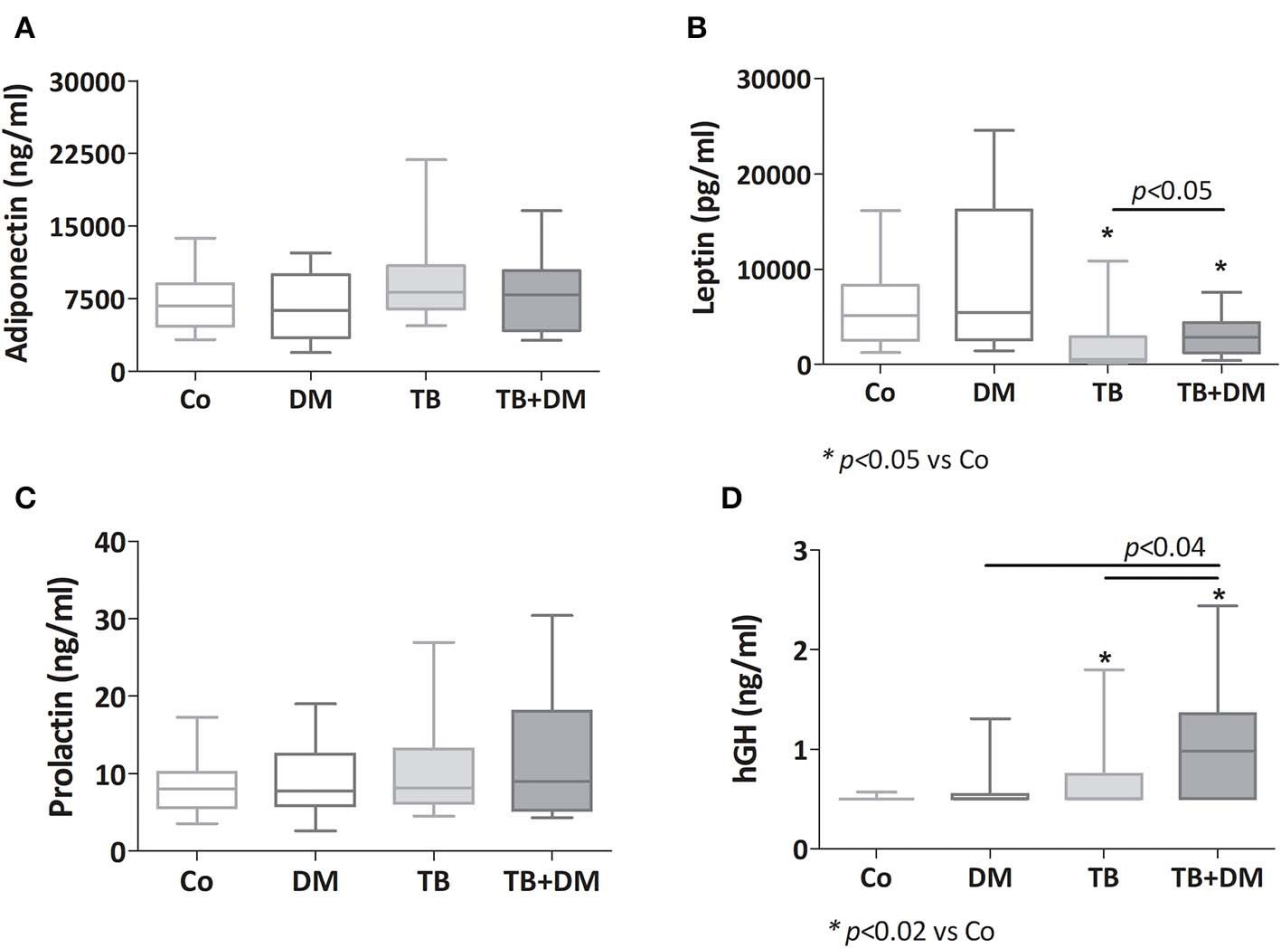
Plasma levels of adiponectin **(A)**, leptin **(B)**, prolactin **(C)** and human growth hormone (hGH, **D**) in controls (Co), patients with type 2 diabetes (DM), with pulmonary tuberculosis (TB) or with TB and DM (TB-DM). Box plots show median values, 25–75 percentiles from data in each group with maximum and minimum values.

While adiponectin and prolactin concentrations showed no differences among study groups (Figures 2A,C, respectively), leptin levels appeared decreased in both TB patient groups, much lower in those without DM (Figure 2B). hGH levels were increased in both TB groups particularly in TB+DM cases (Figure 2D).

An additional analysis by sex revealed that decreased levels of leptin prevailed between both groups of men with TB even more in those cases without DM. Regarding hGH, men with TB+DM were the ones showing significantly increased amounts of this hormone (data not shown).

#### Plasma Levels of Cortisol, DHEA and DHEA-S

**Figure 3:**
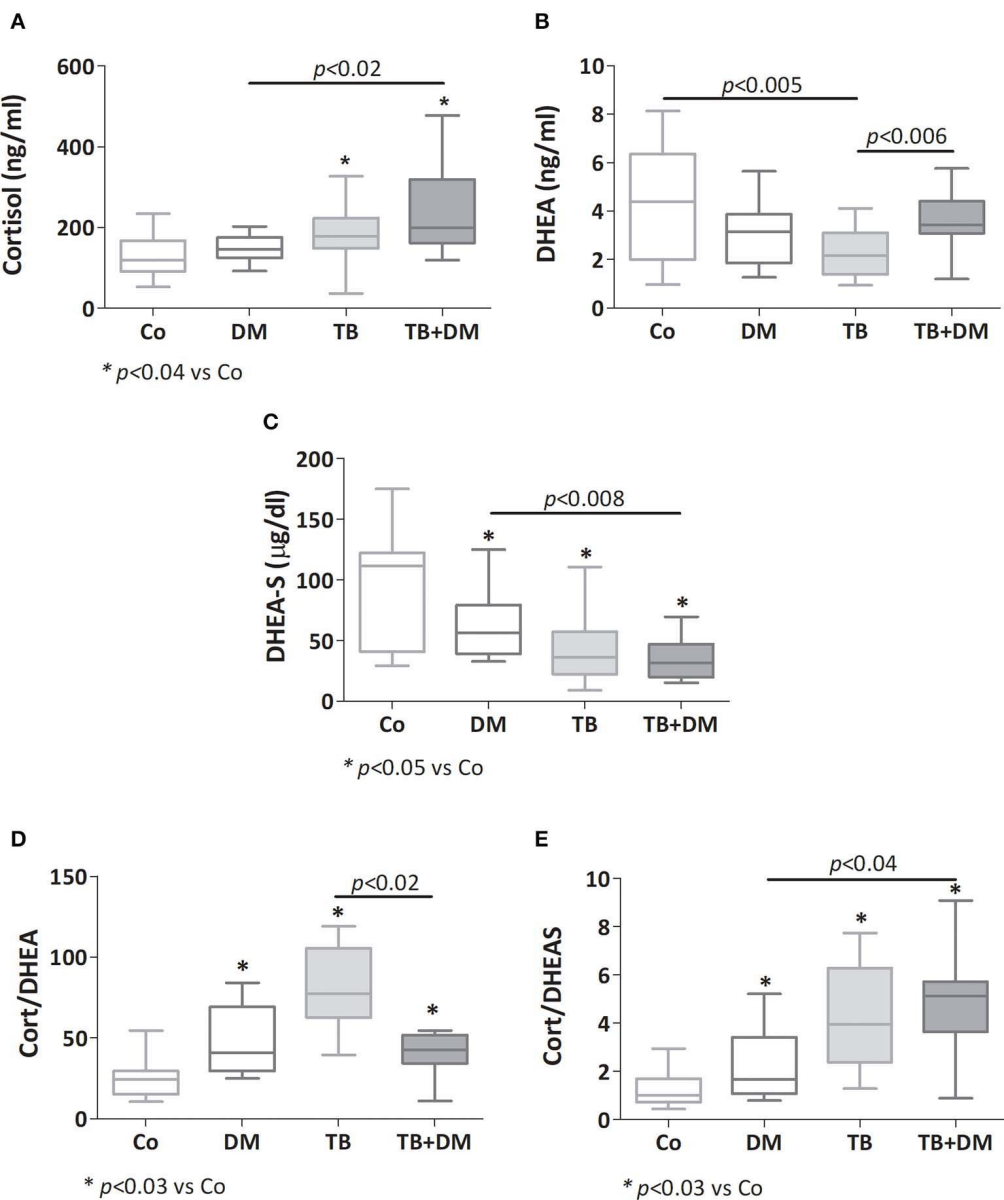
Plasma levels of cortisol **(A)**, dehydroepiandrosterone (DHEA, **B**), dehydroepiandrosterone-sulfate (DHEA-S, **C**), cortisol/DHEA ratio **(D)** and cortisol/DHEA-S ratio **(E)** in controls (Co), patients with type 2 diabetes (DM), with pulmonary tuberculosis (TB) or with TB and DM (TB-DM). Box plots show median values, 25–75 percentiles from data in each group with maximum and minimum values.

As reported earlier, patients with TB had a higher cortisol concentration (^*^p < 0.04 vs. Co, Figure 3A), as did patients with TB+DM (*p* < 0.04 vs. Co and *p* < 0.02 vs. DM). Plasma concentrations of DHEA were significantly lower in TB group compared to Co, but not in patients with TB+DM, who even showed values higher than TB cases (Figure 3B). However, DHEA-S levels were diminished in both groups of TB patients, particularly the TB+DM ones, as well as DM cases (Figure 3C). When analyzing the Cort/DHEA ratio (Figure 3D), this was significantly increased in the three groups of patients compared with those from Co, a bit less pronounced in the TB+DM group. The same was true when comparing the Cort/DHEA-S ratio (Figure 3E), with TB+DM differing in turn from DM.

### Relative Levels of mRNAs Expression for GRα, GRβ and the Enzymes 11βHSD1 and 11βHSD2 in PBMC From the Study Groups

**Figure 4:**
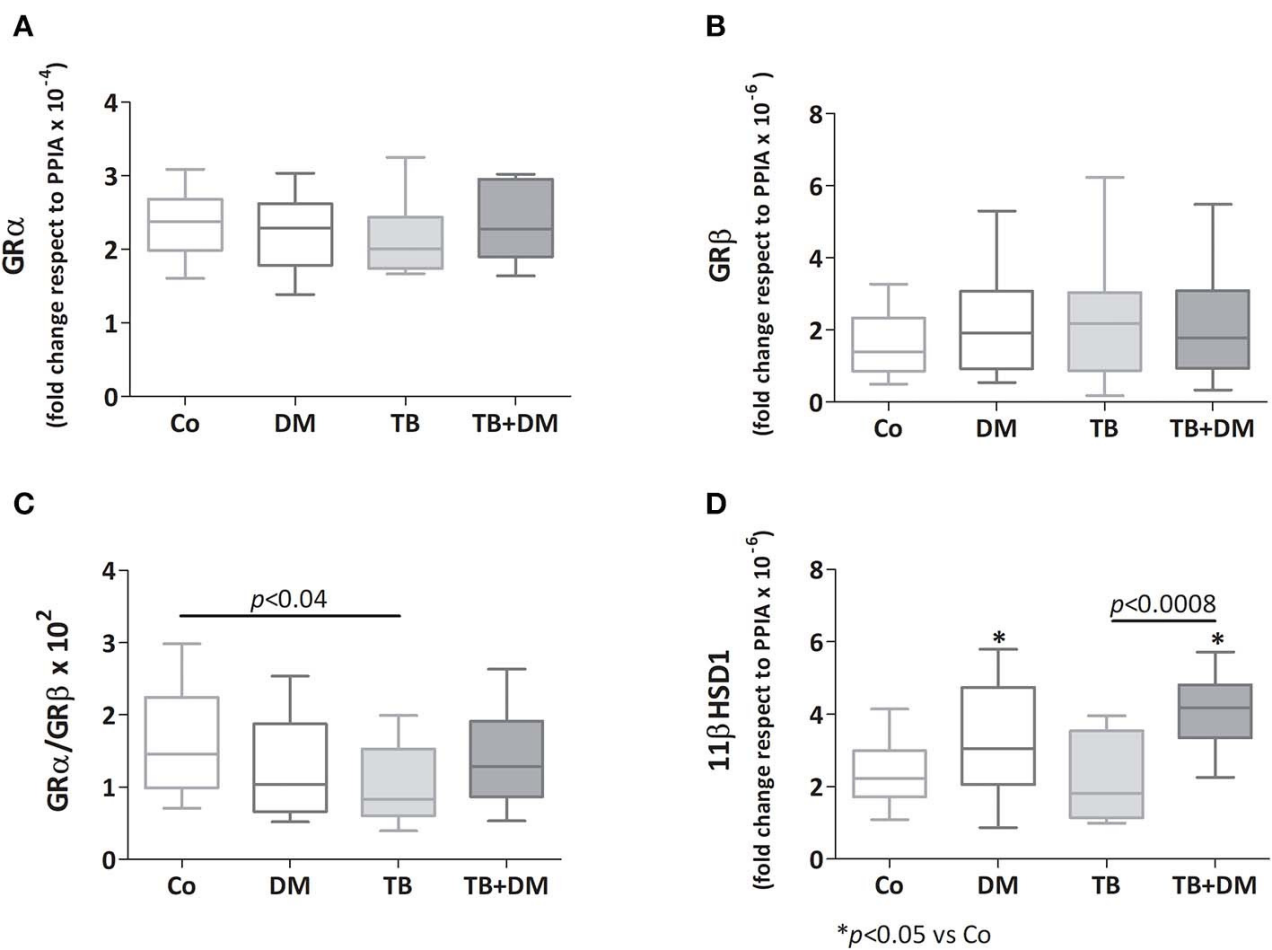
Relative expression of mRNA glucocorticoids receptor (GR) isoforms α (GR α, **A**) and β (GRβ, **B**), 11βHSD1 enzyme **(D)**, and the GRα/GRβ ratio **(C)** in peripheral blood mononuclear cells (PBMC) from controls (Co), patients with type 2 diabetes (DM), with pulmonary tuberculosis (TB) or with TB and DM (TB-DM). Data are expressed as fold change respect to PPIA. Box plots show median values, 25–75 percentiles from data in each group with maximum and minimum values.

As depicted in Figures 4A,B there were no significant differences in transcript expression levels for GRα and GRβ, although the GRα/GRβ ratio was found to be diminished in patients with TB when compared with Co (Figure 4C). Regarding the 11βHSD1 mRNA, its levels were increased in both groups of patients with DM, respect to those of Co, as well as TB+DM patients if compared to TB (Figure 4D). In general terms the 11βHSD2 enzyme was not expressed, or at very low levels, even with optimized reaction conditions (24).

### Lymphoproliferation Studies

**Figure 5:**
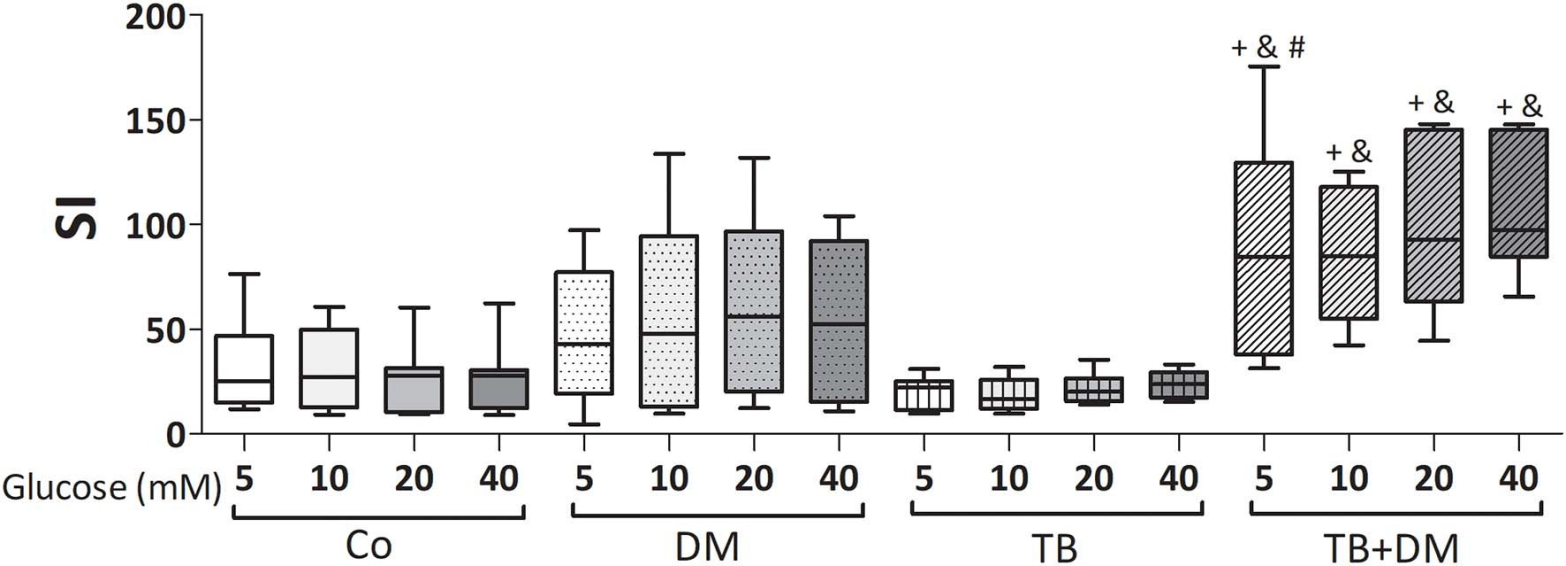
Effects of supraphysiological glucose doses on *Mtbi*-induced proliferation of peripheral blood mononuclear cells (PBMC) from controls (Co), patients with type 2 diabetes (DM), with pulmonary tuberculosis (TB) or with TB and DM (TB-DM). *Mtbi*: γ-irradiated H37Rv *M. tuberculosis* strain. Results are shown as Stimulation index (SI: average of counts per minute -cpm- in *Mtbi* stimulated cultures/average of cpm in unstimulated cultures). Box plots show median values, 25–75 percentiles from data in each group with maximum and minimum values. ^+^different from HCo, *p* < 0.02; ^&^different from TB, *p* < 0.003; ^#^different from DM, *p* < 0.05; in each case when comparing with the same glucose dose.

**Figure 6:**
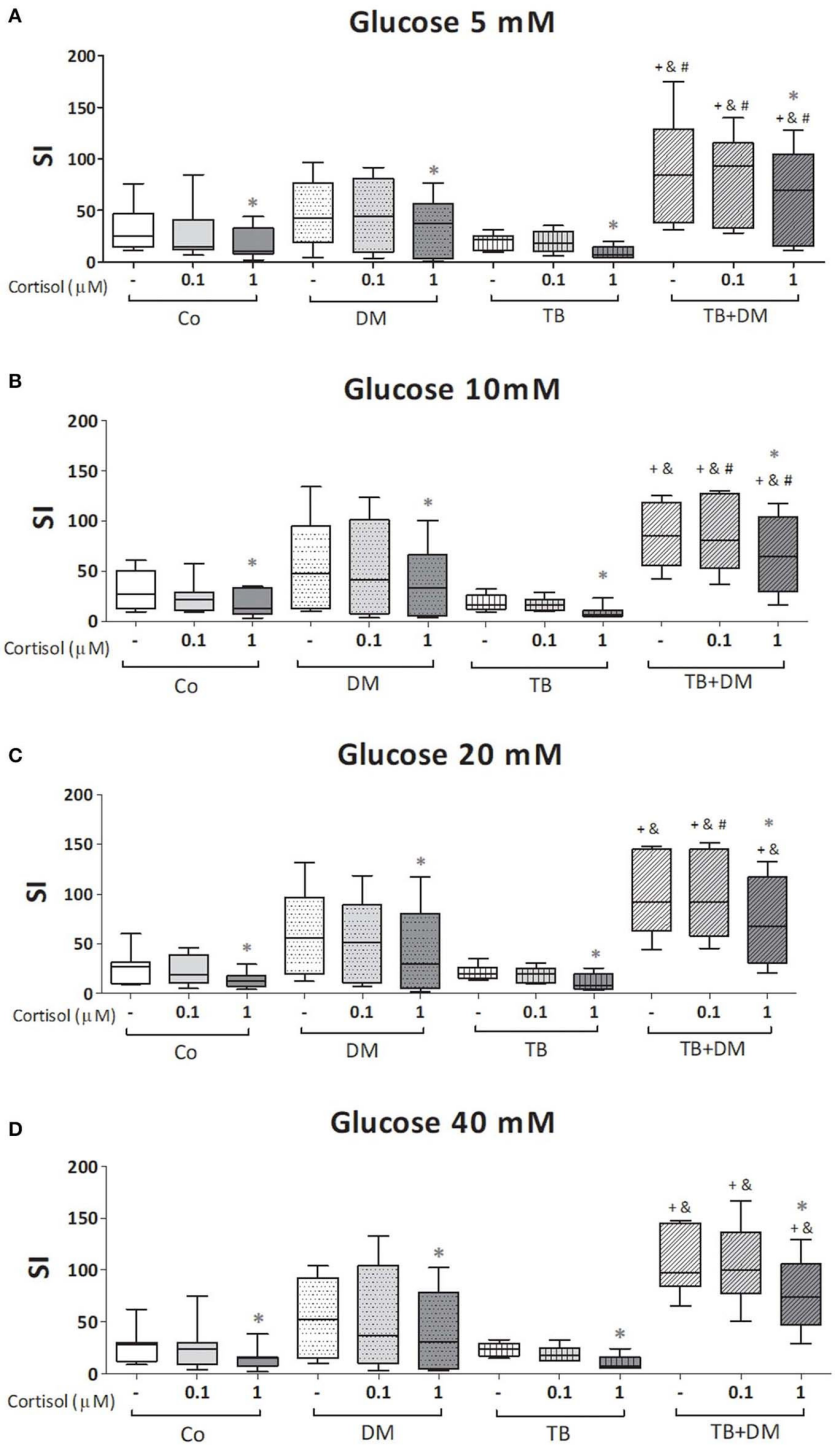
Effects of glucose doses (5 mM, **A**; 10 mM, **B**; 20 mM, **C**; 40 mM, **D**) on cortisol-induced inhibition of *Mtbi-*blastogenesis by peripheral blood mononuclear cells (PBMC) from controls (Co), patients with type 2 diabetes (DM), with pulmonary tuberculosis (TB) or with TB and DM (TB-DM). *Mtbi*: γ-irradiated H37Rv *M. tuberculosis* strain. Results are shown as Stimulation index (SI: average of counts per minute -cpm- in *Mtbi* stimulated cultures/average of cpm in unstimulated cultures). Box plots show median values, 25–75 percentiles from data in each group with maximum and minimum values. ^+^different from Co, *p* < 0.04; ^&^different from TB, *p* < 0.02; ^#^different from DM, in each case when comparing the same treatment between groups, *p* < 0.05; *different from cultures without cortisol and from those treated with 0.1 uM cortisol within the same group, *p* < 0.05.

**Figure 7:**
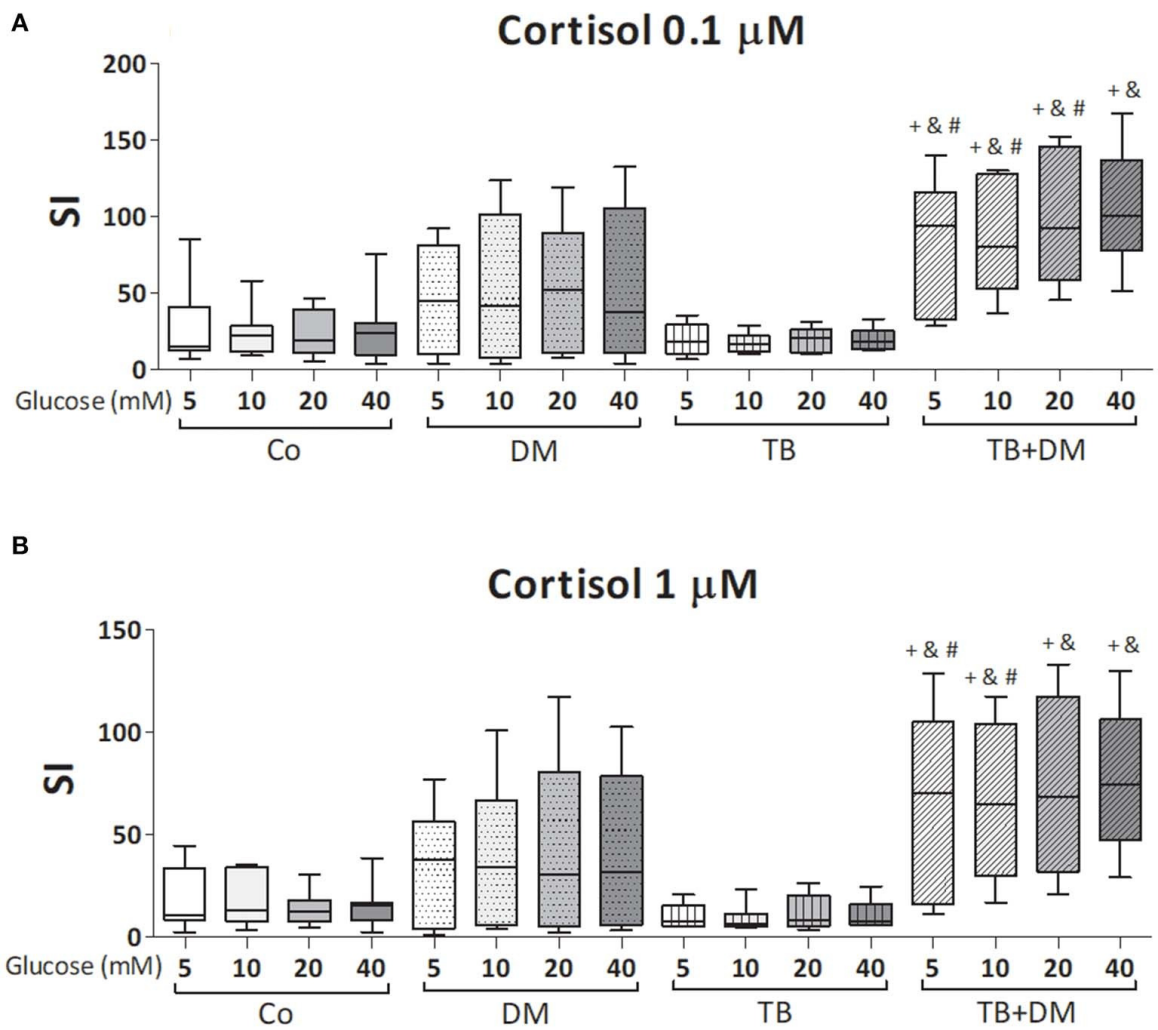
Effects of glucose doses on *Mtbi-*blastogenesis of peripheral blood mononuclear cells (PBMC) from in controls (Co), patients with type 2 diabetes (DM), with pulmonary tuberculosis (TB) or with TB and DM (TB-DM) treated with cortisol 0.1 μM **(A)** or 1 μM **(B)**. *Mtbi*: γ-irradiated H37Rv *M. tuberculosis* strain. Results are shown as Stimulation index (SI: average of counts per minute -cpm- in *Mtbi* stimulated cultures/average of cpm in unstimulated cultures). Box plots show median values, 25–75 percentiles from data in each group with maximum and minimum values. ^+^different from Co, *p* < 0.04; ^&^different from TB, *p* < 0.02; ^#^different from DM, *p* < 0.05; in each case when comparing with the same glucose dose.

The effects of different Glc doses on the proliferative capacity of PBMC against *Mtbi* are shown in Figure 6. Regardless of the Glc dose, the blastogenic response of cells, expressed as Stimulation Index (SI), from each study group showed a similar pattern (Figure 5). For instance, the SI of the TB group was lower than that from Co, and negatively correlated with IL-10 levels (*r* = −0.54, *p* < 0.03), while both groups of DM patients showed the highest responses, in the case of TB+DM statistically significant from TB and Co groups. In essence, increasing Glc concentrations did not modify the *Mtbi*-driven blastogenesis in the four study groups (Figure 5). Data from studies by adding cortisol in presence of different Glc concentrations are summarized in Figure 6. The higher dose of cortisol (1 μM) decreased proliferation regardless of the study group or Glc concentration, being significantly lower than the results obtained when using a lower cortisol dose (0.1 μM; Figures 6A–D). Between group differences in the blastogenic response continued to show the pattern described in Figure 6, regardless of the Glc or cortisol doses (Figures 6A–D). Notably, hyperglycemia did not modify the inhibitory cortisol effect (Figures 7A,B).

### Quantification of Mediators in Culture Supernatants of PBMC

According to the study purposes, 24 h culture supernatants from PBMC subjected to the above described treatments (Materials and Methods section Studies on the *In vitro* Effects of Cortisol and Glucose), were assessed for the levels of proinflammatory (TNF-α, IL-1β, IL-6), and anti-inflammatory (IL-10) cytokines as well as the ones representing the Th1 (IL-2, IFN-γ), Th2 (IL-4) and Th17 (IL-17A) profiles. According to data from the lymphoproliferation studies, we decided to quantify cytokines in six parallel cultures undergoing one of the following stimulation procedures: Glc 5 mM, Glc 20 Mm, Glc 5 mM+ *Mtbi*, Glc 20 Mm+ *Mtbi*, Glc 5 mM + Cortisol 1 μM + *Mtbi*, Glc 20 mM + Cortisol 1 μM + *Mtbi*. All *Mtbi*-stimulated cultures contained increased levels of TNF-α, IL-1β, IL-2, IFN-γ, and IL-10 which remained unmodified by Glc treatment even at the 20 μM dose, except for IL-1β production from PBMC of Co (Table 3). IL-6 levels were largely increased before stimulation, beyond the upper detection limits of the Kit, whereas IL-4 and IL-17A remained undetectable.

**Table 3:** Effect of glucose doses on cytokine production by Mtbi-stimulated peripheral mononuclear cells from the different study groups.

***Mtbi* stimulus**	**Co** **(*****n*** **=** **7)**		***p***	**DM** **(*****n*** **=** **9)**		***p***	**TB** **(*****n*** **=** **7)**		***p***	**TB+DM** **(*****n*** **=** **6)**		***p***
	**Glc 5 mM**	**Glc 20mM**		**Glc 5mM**	**Glc 20mM**		**Glc 5mM**	**Glc 20mM**		**Glc 5mM**	**Glc 20mM**	
TNF-α (pg/ml)	900 (537–1,032)	840 (524–1,239)	n.s.	565 (278–1,729)	876 (35–1,394)	n.s.	1,121 (602–3,298)	1,195 (619–3,086)	n.s.	830 (344–2,904)	1,167 (462–2,877)	n.s.
IL-1β (pg/ml)	4,053 (3,610–5,066)	4,526 (4,153–5,232)	0.03	5,181 (4,516–5,706)	5,286 (4,841–5,857)	n.s.	4,221 (3,776–5,400)	4,746 (4,479–5,579)	n.s.	4,099 (3,706–5,498)	4,302 (3,625–4,754)	n.s.
IL-2 (pg/ml)	17.8 (12,6–29,2)	18,3 (11.2–24.5)	n.s.	10.9 (7.52–32.6)	12,3 (5.99–26.9)	n.s.	16.4 (8.01–38.7)	15.3 (8.13–37.4)	n.s.	91.8 (40.4–323)	85.5 (40.9–254)	n.s.
IFN-γ (pg/ml)	8.72 (6.98–30.6)	9.42 (6.94–34.4)	n.s.	16,0 (3.70–37.4)	11,1 (3,42–48,0)	n.s.	21,5 (12,4–37,4)	22,9 (13.8–43.4)	n.s.	94,2 (21.2–654)	106 (22.5–634)	n.s.
IL-10 (pg/ml)	160 (125–285)	154 (121–253)	n.s.	195 (176–255)	198 (184–243)	n.s.	375 (63.3–490)	380 (66.0–468)	n.s.	208 (50.0–241)	192 (115–262)	n.s.

*Co, controls; DM, patients with type 2 diabetes mellitus; TB, patients with pulmonary tuberculosis; TB+DM, patients with pulmonary tuberculosis and type 2 diabetes mellitus; Glc, glucose; n.s., not significant. Results are shown as median (25–75 percentiles)*.

**Figure 8:**
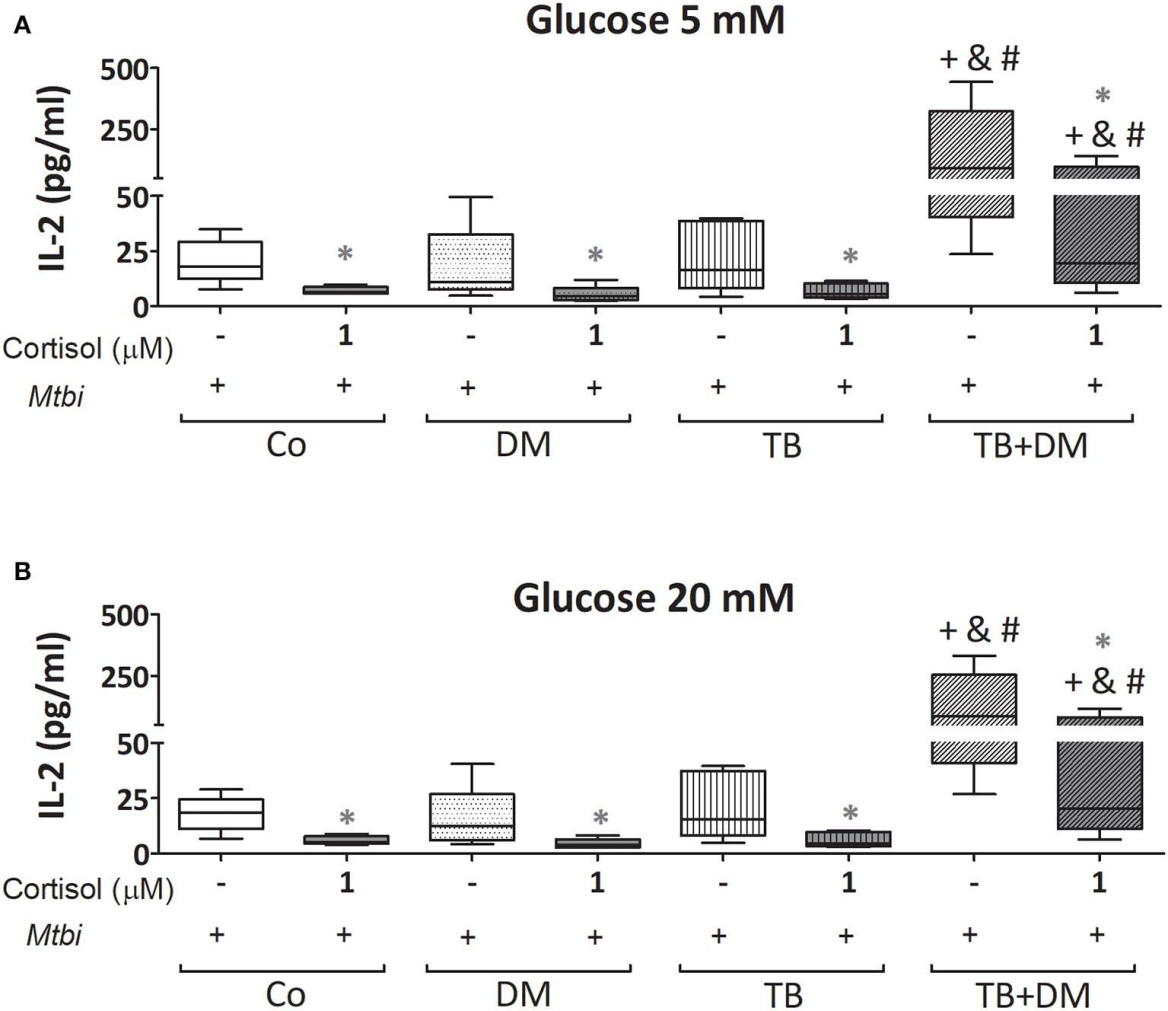
IL-2 production by cultures of peripheral blood mononuclear cells (PBMC) from in controls (Co), patients with type 2 diabetes (DM), with pulmonary tuberculosis (TB) or with TB and DM (TB-DM), stimulated with *Mtbi* and treated with glucose 5 mM **(A)** and 20 mM **(B)** with or without cortisol (1 μM). *Mtbi*: γ-irradiated H37Rv *M. tuberculosis* strain. Box plots show median values, 25–75 percentiles from data in each group with maximum and minimum values. ^+^different from Co, *p* < 0.03; ^&^different from TB, *p* < 0.04; ^#^different from DM, *p* < 0.04; in each case when performing between-group comparisons for the same treatment; *different from cultures without cortisol within the same group. *p* < 0.03.

**Figure 9:**
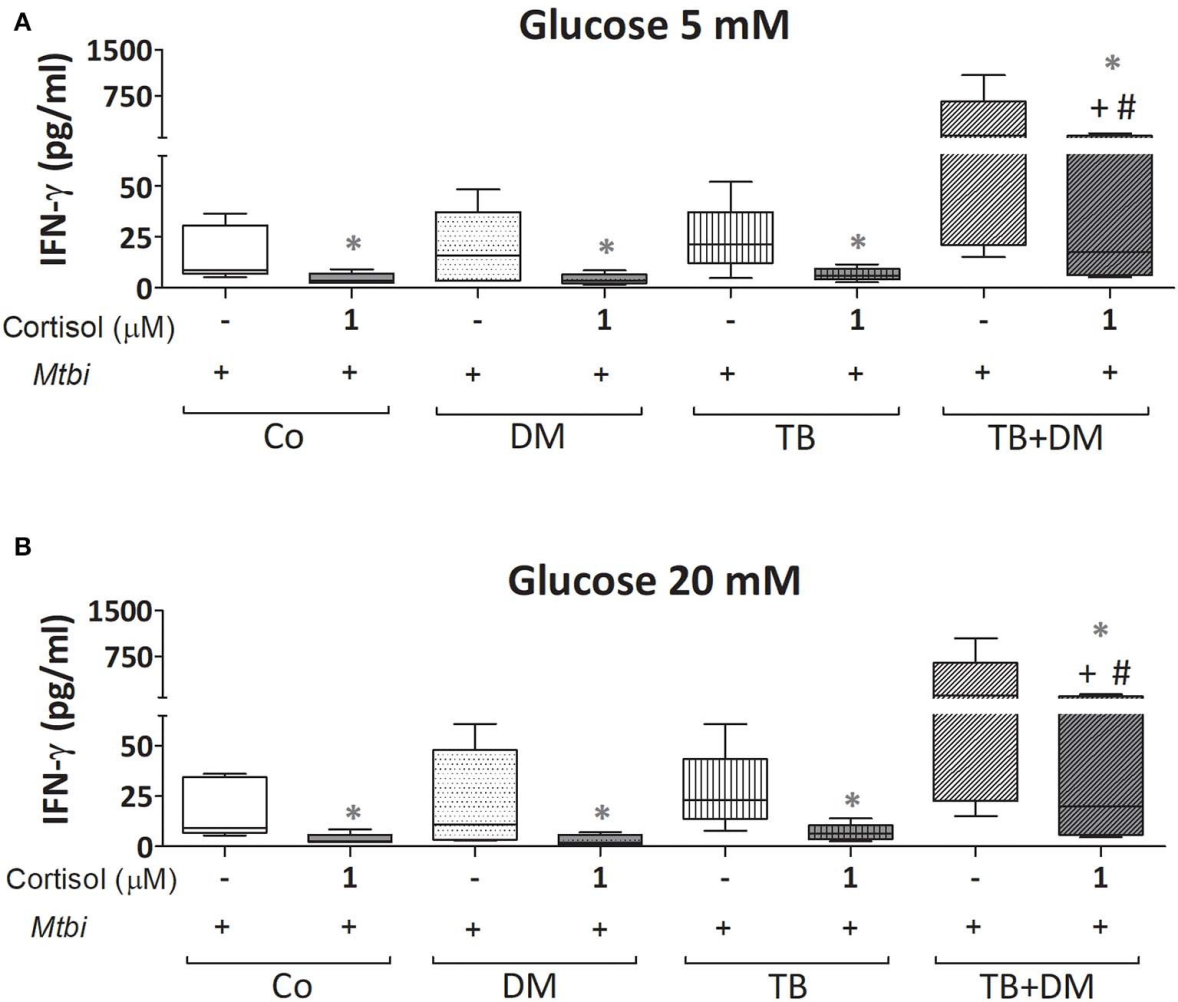
IFN-γ production by cultures of peripheral blood mononuclear cells (PBMC) from controls (Co), patients with type 2 diabetes (DM), with pulmonary tuberculosis (TB) or with TB and DM (TB-DM), stimulated with *Mtbi* and treated with glucose 5 mM **(A)** and 20 mM **(B)** with or without cortisol (1 μM). *Mtbi*: γ-irradiated H37Rv *M. tuberculosis* strain. Box plots show median values, 25–75 percentiles from data in each group with maximum and minimum values. ^+^different from Co, *p* < 0.04; ^#^different from DM, *p* < 0.05; in each case when performing between-group comparisons for the same treatment; *different from cultures without cortisol within the same group *p* < 0.03.

When comparing cytokine production, cultured PBMC of patients with TB+DM had the highest levels of IL-2 (Figure 8) and IFN-γ (Figure 9), in the case of IL-2 statistically different from the remaining groups (Figures 8A,B). Levels of TNF-α, IL-1β, and IL-10, in stimulated cultures were similar for all study groups (data not shown). Cortisol treatment decreased the production of all above described cytokines, regardless of subject groups or Glc doses (Figures 8, 9). As regards to IL-2 (Figures 8A,B), although their levels dropped, they remained significantly elevated in the TB+DM group respect to the remaining groups. The same was true when analyzing IFN-γ concentrations, although differences were only significant in relation to Co and DM (Figures 9A,B).

## Discussion

TB-DM comorbidity became more relevant in recent decades due to the diabetic population marked increase; particularly in low and middle-income countries, where PTB is prevalent. Both pathologies present, by themselves, alterations in the bidirectional communication between the immune and neuroendocrine systems, with an important impact on the metabolic component (25–27).

Our work evidenced that both groups of PTB patients presented an important inflammatory response, reflected in high systemic levels of IL-6, CRP, and IFN-γ. Several reports indicate a greater increase of proinflammatory mediators in PTB patients suffering DM compared to non-diabetic patients (28, 29). This may reinforced by the increased presence of hGH in plasma from TB+DM cases, considering that this hormone exerts a contributory role for the development of the IR and the accompanying inflammation (30, 31).

As in previous studies (32–34), patients with only TB showed decreased blastogenesis, which in turn correlated negatively with IL-10 levels, a cytokine of recognized inhibitory effect on lymphoproliferation (35). Such decreased specific proliferation may be partly attributed to a recruitment of cells committed toward the site of the lesion (19). This mechanism seems to be altered in diabetic patients with active PTB (27, 36) and could to account for the increased *Mtb*-driven mitogenesis of PBMC.

The systemic increase of IL-10 from TB+DM patients, also reported by Kumar and collaborators (37), may be mirroring a regulatory mechanism of IR, given its well-known anti-inflammatory and anti-proliferative effects (35, 38). At the same time, IL-10 seems to be detrimental in mouse and human TB, as it favors mycobacterial survival in macrophages by inhibiting phagosome maturation, reducing NO production (39) and in turn blocking IFN-γ (40, 41) signaling. Some studies suggest that IL-10 would be a marker of disease progression (42–46). In our cases, analysis according to disease severity showed a significant increase of IL-10 in TB+DM patients with progressive pulmonary involvement (data not shown).

Notably, IL-10 levels were also augmented in DM patients who also displayed increased amounts of CRP together with a high ESR resembling some sort of pro- and anti-inflammatory influences.

As seen in former studies (33, 34, 47, 48), the unbalanced relationship between steroid hormones recorded in TB patients, increased and decreased levels of cortisol and DHEA-DHEA-S, respectively, was also found in TB+DM patients. This may be explained by assuming that the adrenal gland is trying to preserve cortisol production at the expense of DHEA synthesis, to counteract the inflammatory response accompanying active disease. Decreased levels of DHEA and DHEA-S, may be detrimental in TB, since they preferentially favor the Th1 profile of the IR in addition to exerting anti-inflammatory activities (49–52). DHEA-S is not bioactive; but constitutes the natural reservoir of DHEA being therefore a stable marker of its availability.

As regards GR isoforms, while high GC-responsiveness would typically reduce GRα increasing GRβ and therefore dampening GC effects, this was not so evident in TB, probably because of its chronic nature and several endocrine alterations. The reduced GRα/GRβ ratio seen in TB patients is compatible with a certain degree of resistance to GC endogenous function. In a recent study, Martins et al. reported that GRβ expression levels were dramatically increased in PBMC of patients with the metabolic syndrome compared to lean controls (53). Our present lack of GRβ transcript alterations in control subjects may be explained by considering that their BMI situated below the ones displayed by the group of dysmetabolic Brazilian patients. Mouse studies revealed that GRβ causes higher glucose levels along with and increased immune-inflammatory response (54), but in our hands GRβ expression was not associated to any particular change in the profile of metabolic or immune-endocrine mediators. Despite TB+DM cases showed no differences in the GRα/GRβ ratio, transcriptional levels of 11βHSD1, which favors the availability of cortisol at the cellular level, were clearly elevated in both groups of DM patients. The fact that TB+DM patients presented high levels of plasma cortisol, along with 11βHSD1 transcripts and IFN-γ values points out to some degree of HPA axis dysfunction and/or GC resistance in them (55, 56).

The lower BMI from TB patients is in line with our former demonstrations in this regard, in which we also documented a negative association between BMI and IL-6 levels, probably reflecting the high-energy demand required to support the chronic inflammatory response (48, 57). In the case of TB+DM patients they exhibit a negative association between cortisol levels and BMI (data not shown). With regard to TB+DM patients, 60% of them fell within the overweight category in line with some evidence indicating an association between overweight and obesity with an increased risk for DM and pre-DM development (58–60). While 77% of individuals from the Co group had a high BMI, our findings are in line with a report from WHO (GLOBAL STATUS REPORT on non-communicable diseases 2014), referring to an increase in BMI in most developing countries between the 2010–2014 period, in our country from 27.2 to 27.8 (61).

Present results in TB patients are consistent with our earlier studies (48), revealing an orexigenic pattern characterized by low and high levels of leptin and adiponectin, respectively. This not being the case of TB+DM patients in whom leptin levels appeared diminished to a lesser extent, with no changes in adiponectin concentrations. Reduced levels of leptin in both groups of patients with TB may be partly due to the high systemic amounts of proinflammatory mediators, known to suppress leptin synthesis (62, 63). The different pattern seen in TB+DM patients may have to do with their preserved or even increased BMI, implying a greater adiposity. Likewise, leptin has been reported as facilitating the cellular response, particularly that of the Th1 profile (64). Adiponectin was found reduced in dysregulated diabetic patients, compared to slim controls (65), but in our study we found differences in this regard. Between study differences may be explained by considering that most DM patients were being treated for their diabetes, and their BMI was comparable to the one recorded in Co group.

The inflammatory component observed in DM and TB+DM patients is likely to account for their insulin resistance, mainly in the former group (66–68). Decreased amounts of both androgens (DHEA and DHEA-S) may be also implied in insulin resistance, since they favor body weight (69, 70) and adipose tissue reduction (71) while stimulating Glc uptake (70, 72).

Moving to the immune-metabolic communication, innate and adaptive immune cells must respond rapidly in the presence of noxious stimuli, for which they must drastically alter their metabolism to achieve a high rate of cell division, as well as the synthesis and secretion of various molecules necessary for the development of a protective IR. While leukocytes use several types of energy sources (fatty acids, cholesterol, vitamins, trace elements, amino acids, monosaccharides), Glc and glutamine emerge as the main metabolic substrates, representing 70% of energy sources (73).

Studies assessing the immune status of DM patients against several microbial antigens showed a dysfunctionality of innate and adaptive cells, particularly in cases with chronic hyperglycemia (high HbA1c levels) (14, 74). Such alterations appeared to reverse under a proper glycemic control (14, 75–77), although transient and chronic hyperglycemia induce epigenetic modifications, known under the term “metabolic memory” likely to influence substantially immunocompetent cell activity (78, 79).

In the present study, *Mtbi*-stimulated PBMCs from TB+DM patients showed the highest mitogenic response together with an abundant production of IL-2 and IFN-γ. Present results are in line with the study by Restrepo et al. showing a predominant Th1 profile upon PPD stimulation of PBMC from patients with this comorbidity (28). Kumar et al. reported the presence of a Th1/Th17 profile when studying cells from TB+DM cases (80), while Stalenhoef et al. found no differences in IFN-γ production levels when comparing TB patients with or without DM (81). Such dissimilarities may be due to ethnical differences, control status of DM and experimental conditions, among others.

In line with the well-known immunostimulant effects of IL-2 (82), in our case IL-2 levels were positively associated with *Mtb*-driven proliferation in DM and Co individuals, as well as with IFN-γ in all study groups. The increased *in vitro* synthesis of IFN-γ by stimulated PBMC from TB+DM patients was paralleled by augmented amounts of this cytokine in circulation. While being critical for mycobacterial elimination (83, 84), IFN-γ also exerts proinflammatory effects likely to mediate tissue damage when improperly regulated. As such, and over-expansion of cells with a Th1 profile may constitute a double-edged sword in the context of TB+DM.

Hyperglycemia can lead to an inflammatory state through several pathways (85–87) with advanced glycation end products playing a substantial role is this regard. These products, which are abundant in uncontrolled diabetic patients, promote inflammation not only by activating NF-kB (88) but also by working in combination with other inflammatory mediators like the high mobility group box 1 protein to aggravate the inflammatory process (89, 90). Consequently, DM individuals with uncontrolled hyperglycemia are likely to show a greater inflammatory and lymphoproliferative response upon exposure to an infectious agent, increasing tissue damage as well. In our hands, both groups of DM patients had the greatest SI, statistically significant in TB+DM, who in turn showed an increased IL-2 and IFN-γ production.

*In vitro* studies have shown an increased release of inflammatory cytokines by PBMC from healthy individuals when exposed to supraphysiological doses of Glc (91, 92). Lachmandas et al. observed that PBMC from healthy people produced higher levels of TNF-α, IL-1β, and IL-6 before stimulation in a hyperglycemic microenvironment, without changes in the levels of IFN-γ, IL-17A, and IL-22, suggesting that Glc would have a greater effect on monocytes than in lymphocytes (18, 83, 84).

In our case, specific stimulation under a hyperglycemic scenario led to an increased IL-1β production only in Co cells, with the specific lymphoproliferation and production of other mediators being unmodified no matter the study groups. Differences in technical approaches, i.e., stimulation procedures and time point evaluations, may account for inconsistencies between present studies and former reports.

Our former demonstration that cortisol treatment, at doses resembling an acute stress situation, inhibited the specific proliferative response and synthesis of IFN-γ by PBMC of TB patients and Co (19) along with the increased systemic levels of cortisol of TB+DM patients, prompted us to analyze cortisol effects in them. As seen in TB patients, high cortisol doses inhibited proliferative capacity and production of TNF-α, IL-1β, IL-2, IFN-γ, and IL-10, in TB+DM and DM patients as well as Co. Although TB+DM continued to show a remarkable response despite this steroid treatment. Cortisol inhibitory effects on pro-inflammatory cytokine production are achieved by several transcriptional repression mechanisms (93–97), with some inconsistencies as to its role in the production of anti-inflammatory mediators in light of evidence reporting a stimulating or inhibitory influences on IL-10 *in vitro* synthesis (98–102). Whether the present diminished IL-10 production is to some extent related to the decreased TNF-α and IFN-γ synthesis, which by themselves may promote IL-10 production (103), remains to be established.

Although the effects of Glc and cortisol on IR have been extensively analyzed, their combined effect on PBMC had not been studied so far. As shown, high Glc doses did not modify the cortisol-induced inhibition on mitogenesis, nor cytokine production from different study groups.

The bulk of presented results points out that the deregulated immune-endocrine-metabolic status from DM patients becomes more pronounced in those with the TB comorbidity. This is supported not only by the further increased systemic proinflammatory response of TB+DM patients but also for the demonstration that PBMC are more likely to develop an exacerbated response against *Mtb*, reflected in a more pronounced Th1 profile. A better control of the diabetic status will promote a more favorable course of TB in the context of TB+DM comorbidity.

## Data Availability Statement

All datasets generated for this study are included in the article/supplementary material.

## Ethics Statement

The studies involving human participants were reviewed and approved by The Bioethical Committee of the School of Medical Sciences, National University of Rosario. The patients/participants provided their written informed consent to participate in this study.

## Author Contributions

RF, AD, BB, GG, and LD'A designed and carried out experimental procedures. DB, WG, SL, YB, and RG selected voluntaries and performed blood samples extraction. RF, LD'A, MB, and OB performed data analysis and wrote the paper.

### Conflict of Interest

The authors declare that the research was conducted in the absence of any commercial or financial relationships that could be construed as a potential conflict of interest.
